# Supplementary material for: Motor biases reflect a misalignment between visual and proprioceptive reference frames
Source: eLife. 2026 Feb 19;13:RP100715. doi: 10.7554/eLife.100715 (PMC12919971; doi:10.7554/eLife.100715)
Supplement: Supplementary file 1. — See ‘Methods’ for description of each parameter. (a) Participant moved on the trackpad in Exp 3b. We assumed the movement distance was 1 cm and scaled the parameters accordingly. (b) The estimate of yr\begin{document}$y_{r}$\end{document} is much smaller in Exp 3b compared to Exp 1b, suggesting the workspace in Exp 3b is closer to the body. This attenuates the average magnitude of the bias. [file elife-100715-supp1.docx]

| Model (Exp) | $\boldsymbol{x}_{\boldsymbol{r}}$(cm) | $\boldsymbol{y}_{\boldsymbol{r}}$(cm) | $\boldsymbol{x}_{\boldsymbol{e}}$ | $\boldsymbol{y}_{\boldsymbol{e}}$ | $\boldsymbol{a(^{\circ})}$ | $\boldsymbol{b(^{\circ})}$ |
| --- | --- | --- | --- | --- | --- | --- |
| TR+TG (1b) | 47.8 | -11.2 | -0.25 | -0.05 | 19.0 | 3.9 |
| TR+TG (3b) | 96.6 | -0.002 | -0.30 | -0.00 | 19.0 | 1.4 |
| Trans. (1b) | 60.5 | -12.8 | -0.27 | -0.06 |  |  |
| Trans. (3b) | -0.15 | 17.3 | -0.004 | 0.03 |  |  |
| TG (1b) |  |  |  |  | 15.0 | 4.6 |
| TG (3b) |  |  |  |  | 15.5 | 3.6 |
|  | $x_{e}$(cm) | $y_{e}$(cm) |  |  |  |  |
| PropV+ TG (1b) | 0.26 | 0.00 |  |  | 12.0 | 4.5 |
| PropV+ TG (3b) | -0.19 | -0.41 |  |  | 6.4 | 0.01 |
| PropV (1b) | 0.20 | 0.00 |  |  |  |  |
| PropV (3b) | 0.40 | 0.00 |  |  |  |  |
|  | $\boldsymbol{x}_{\boldsymbol{0}}\boldsymbol{(^{\circ})}$ | $\boldsymbol{y}_{\boldsymbol{0}}\boldsymbol{(^{\circ})}$ | $\boldsymbol{x}_{\boldsymbol{e}}\boldsymbol{(^{\circ})}$ | $\boldsymbol{y}_{\boldsymbol{e}}\boldsymbol{(^{\circ})}$ |  |  |
| PropJ+TG (1b) | 42.6 | 110.3 | 0.73 | 0.06 | 6.1 | 1.3 |
| PropJ+TG (3b) | 89.7 | 156.8 | 0.04 | -0.15 | 7.6 | 1.4 |
| PropJ (1b) | 71.9 | 119.1 | 0.00 | 0.01 |  |  |
| PropJ (3b) | 89.7 | 168.0 | -0.002 | -0.13 |  |  |
